# 

**Published:** 1876-12

**Authors:** 


### CONTENTS:

ARTICLE I.—Plastic Filling. By S. P. Cutler, M. D., D. D. S. .......... 337

ARTICLE II.—Transplantation and Replantation of Teeth. By William Taft, D. D. S. 344

ARTICLE III—Resetting and Transplanting Teeth. By H. N. Wadsworth, D. D. S. 354

ARTICLE IV.—Finishing Fillings. By H. Judd, M. D., D. D. S. .......... 360

ARTICLE V.—On the Profession of Dental Surgery. By John Tomes, F. R. S. ..... 366

ARTICLE VI.—Value of Phosphate of Lime as a Therapeutic Agent .......... 370

ARTICLE VII.—Washing and Squeezing Amalgams .......... 372

ARTICLE VIII.—Epithelioma of Face .......... 373

### EDITORIAL, ETC.

The Physiological Action of Alcohol .......... 374
Treatment of Fractured Teeth .......... 375

### BIBLIOGRAPHICAL.

A Manual of Dental Anatomy, Human and Comparative .......... 376
A History of Dental and Oral Science in America .......... 377

### MONTHLY SUMMARY.

Differences between Anæsthesia of Ether and Chloroform .......... 378
Physiology of the Nerve Centres .......... 379
Headache from Eye-Strain .......... 380
Obliteration of Depressed Cicatrices .......... 380
Advance in Dental Surgery .......... 380
Ulceration of Fraenum Linguae .......... 381
Inflammatory Process—Its Nature .......... 381
Composition of the Human Body .......... 382
Cement for Rubber and Glass .......... 382
Influence of Age of Mother on Sex .......... 382
Death from Ether .......... 383
Sulphide of Calcium .......... 383
Mercurial Poisoning from Canned Meat .......... 384
Poisonous Properties of Glycerine .......... 384

